# Clinical relevance of receptor conversion in metastatic breast cancer: Case report

**DOI:** 10.1097/MD.0000000000029136

**Published:** 2022-06-10

**Authors:** Diogo J. Silva, Gonçalo Miranda, Alexandra Mesquita

**Affiliations:** a Medical Oncology Department, Local Health Unity Matosinhos–Hospital Pedro Hispano, Porto, Portugal; b Pathology Department, Local Health Unity Matosinhos–Hospital Pedro Hispano, Porto, Portugal.

**Keywords:** breast cancer, case report, heterogeneity, olaparib

## Abstract

**Introduction:**

Breast cancer comprises several different pathological entities defined by the presence or absence of hormonal receptors and human epidermal growth factor receptor 2 (HER2). During the disease course, the increase in tumor heterogeneity contributes to the discordant expression of estrogen/progesterone receptors and HER2 status between primary and metastatic lesions. We describe a case that demonstrates the clinical relevance of molecular reassessment during metastatic breast cancer progression.

**Patient concerns:**

A 40-year-old Caucasian woman with germline breast cancer gene mutation was referred to a general surgery appointment after breast ultrasound revealed a suspicious nodular lesion in 2012.

**Diagnosis:**

Ultrasound-guided microbiopsy revealed an invasive ductal carcinoma of no special type, hormone receptor-positive, and HER2-negative.

**Interventions:**

The patient underwent modified radical left mastectomy, adjuvant radiotherapy, chemotherapy, and endocrine therapy. Four years after the diagnosis, HER2 positive lung progression was documented, and the patient received anti-HER2 targeted systemic therapy for 15 months. New disease progression with a triple-negative profile was found, and palliative systemic treatment was changed to carboplatin for 3 months until new progression. Based on the results of the OlympiAD trial, monotherapy with Olaparib 300 mg twice daily for 28 days was initiated.

**Outcomes:**

After seven cycles of treatment, patient showed progressive improvement in quality of life and maintained stable disease without significant adverse events.

**Conclusion:**

The clinical relevance of hormone receptor and HER2 status discordance between primary tumors and metastatic lesions has been studied in recent years. This case report illustrates the clinical impact of molecular changes during disease progression and the adaptation of treatment options. This allows for an increase in both survival and quality of life in patients with metastatic breast cancer.

## 1 Introduction

According to Globocan 2020, 2.2 million women were diagnosed with breast cancer and almost 690 000 died from this type of cancer. Over the last 15 years, molecular heterogeneity of breast cancer has been thoroughly described allowing for a better understanding of its biological behaviour and development of molecular-targeted therapies.^[1]^ This molecular knowledge led to a re-classification of breast cancer in 4 subtypes (luminal A, luminal B, basal like and epidermal growth factor 2 positive), shifting clinical management from tumour burden to biology-centred strategies.^[2]^ Currently, clinical management of breast cancer relies on five histological and molecular subtypes used to define the best treatment plan: Triple Negative (oestrogen receptor [ER], progesterone receptor [PR], and human epidermal growth factor receptor 2 [HER2] negative); Luminal A (strongly ER+, PR+, HER2−); Luminal B (ER+, RP+, HER2−, high Ki-67 index), HER2 enriched (ER−, PR− HER-2+); Luminal B HER2+ (ER+, RP+, HER2+ high Ki-67 index).^[3]^

Breast cancer tumor heterogeneity is also a key factor that influences drug resistance and metastatic progression. This feature implies that disease progression should be confirmed by biopsy of metastatic lesions and biological markers reassessed at least once in a metastatic setting.^[4]^ Breast cancer gene (BRCA) germline mutation and phosphatidylinositol-4,5-bisphosphate 3-kinase catalytic subunit alpha somatic mutation status, reassessment of human epidermal growth receptor 2, estrogen receptor, and programmed death-ligand 1 positivity are important factors to define whether targeted therapies can be used. Furthermore, the presence of BRCA germline mutations constitutes a therapeutic target and predicts sensitivity to a specific group of cytotoxic agents.^[5]^

The reported case illustrates the clinical relevance of the full molecular characterization of breast cancer upon progression and its impact on treatment planning.

## 2 Case report

A 40-year-old Caucasian woman, Eastern Cooperative Oncology Group 0, with a familial history of pancreatic and gastric cancer was referred to a general surgery appointment after a breast ultrasound revealed a suspicious nodular lesion with internal calcifications in the lower outer quadrant of the left breast in 2012. Breast and axillary ultrasound-guided microbiopsy demonstrated the presence of invasive ductal carcinoma of no special type hormone receptor-positive and HER2 negative. Thoracoabdominopelvic computed tomography and bone scintigraphy excluded metastatic spread. Regarding patient preference, a modified radical left mastectomy was performed in May/2012 with an anatomopathological report showing the presence of ductal invasive carcinoma with positive hormonal receptors, HER2-negative status, and proliferative index (Ki67) of 18% – pT2N2M0 according to the 8th edition of American Joint Committee on Cancer. Following surgery, the patient received adjuvant chemotherapy with three cycles of fluorouracil (500 mg/m^2^), epirubicin (100 mg/m^2^), and cyclophosphamide (500 mg/m^2^), plus three cycles of docetaxel 100 mg/m^2^ 5 fluorouracil, epirubicin and cyclophosphamide (FEC) plus docetaxel (T), adjuvant radiotherapy, and adjuvant endocrine therapy with tamoxifen plus an human luteinizing hormone-releasing hormone analog. In March/2015, following complaints of increasing asthenia and dyspnea, a thoracic computed tomography (CT) scan revealed the presence of multiple bilateral lung micronodules, indicating lung metastases. Regarding disease progression without evidence of visceral crisis and hormonal status consistent with postmenopausal status, adjuvant endocrine therapy was suspended, and first-line metastatic endocrine therapy with fulvestrant was initiated.

**Figure 1:**
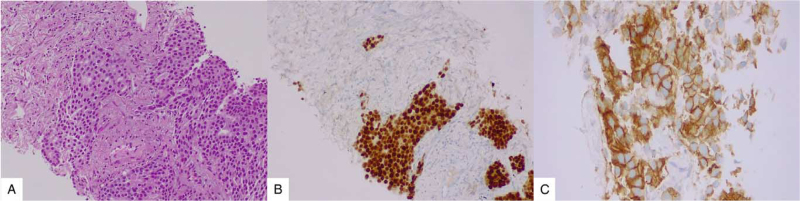
Metastatic breast cancer in lung parenchyma. The lesions are composed by groups of large cells with pleomorphic nuclei. (A–C) Metastatic disease, biopsy performed in 2020 (A, 200×). The malignant cells were strongly immunoreactive for ER (C, 200×) and HER2 (E, 400×). ER = estrogen receptor, HER2 = human epidermal growth factor receptor 2.

Seven months later, reassessment with bone scintigraphy due to back pain revealed an osteoblastic lesion in the lower portion of the left iliac bone. This lesion was further confirmed by a CT scan of both sacroiliac joints, which showed the presence of another osteoblastic lesion on the upper part of the right iliac bone. Hormonal status was reassessed as being compatible with premenopausal status. Endocrine therapy was changed to an aromatase inhibitor plus an human luteinizing hormone-releasing hormone analog associated with biphosphonates until January 2020. After 4 years of stable disease, thoraco-abdominopelvic computed tomography scan showed lung progression with pulmonary biopsy consistent with breast carcinoma hormone receptor-positive and strong positivity for HER2, reviewed by two different pathologists, and confirmed by fluorescent in situ hybridization (Fig. 1). Genetic testing revealed the presence of BRAC2 germline mutations.

**Figure 2:**
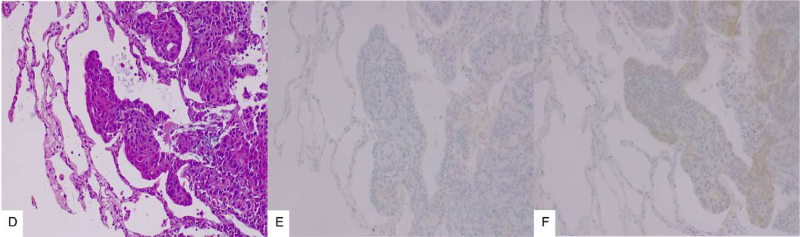
Metastatic breast cancer in lung parenchyma. The lesions are composed by groups of large cells with pleomorphic nuclei. (D–F) Metatastic diasease, biopsy performed in 2021 (B, 200×). The cells were negative for ER (D, 200×), HER2 (F, 200×), and transcription termination factor 1. ER = estrogen receptor, HER2 = human epidermal growth factor receptor 2.

Considering lung progression with a discordant immunohistochemical profile and the benefits associated with HER2 targeted therapy, palliative chemotherapy with docetaxel plus trastuzumab and pertuzumab was proposed after multidisciplinary team meeting approvals. After six cycles of docetaxel plus trastuzumab and pertuzumab for stable disease, maintenance with trastuzumab plus pertuzumab and letrozole was started in June/2020. After six cycles, reassessment revealed lung and bone progression. After a multidisciplinary team meeting, palliative chemotherapy was changed to Ado-trastuzumab emtansine (TDM-1), with lung and ganglionic progression after four cycles. Concerning the tumoral heterogeneity observed during disease progression with HER2 positivity and scarce response to HER2 targeted therapy, a new lung biopsy was performed to re-evaluate the molecular characteristics. Histological and immunohistochemical analyses confirmed the presence of triple-negative breast cancer lung metastasis (Fig. 2).

Documentation of tumoral shift from HER2 positive to triple negative metastatic breast cancer in a woman with a BRCA2 germline mutation led to a multidisciplinary discussion to define further treatment strategies.

Regarding the results of the TNT phase III clinical trial^[6]^ for patients with advanced germline BRCA-mutated triple negative breast cancer, patients were proposed to receive monotherapy with carboplatin with an area under the curve of 6. After four cycles of monotherapy with carboplatin area under the curve 6, reassessment showed lung and bone progression despite the stability of ganglionic disease in June/2021. Regarding new disease progression, patients were eligible for treatment with poly (ADP-ribose) polymerase inhibitors (iPARP) according to the results of the OlympiAD phase III clinical trial.^[7]^ After multidisciplinary team meetings and patient agreement, authorization for the use of olaparib 300 mg twice daily was solicited to the Pharmacy and Therapeutics committee and to the National Authority of Medicines and Health Products. After full approval from the regulatory entities, the patient was administered olaparib (300 mg) twice daily in July/2021. After seven cycles of olaparib 300 mg twice daily, the patient remained stable without any significant adverse events. Furthermore, progressive improvement in the quality of life has been noted since the beginning of PARPi.

## 3 Discussion

Breast cancer results from a diversity of genetic alterations in mammary epithelial cells, leading to a highly heterogeneous disease.^[8]^ current classification of breast cancer is based on the histopathology of the primary tumor, expression of hormone receptors, epidermal growth factor receptor, and genomic profile.

Breast cancer heterogeneity can be divided into “intertumoral heterogeneity” and “intratumoral heterogeneity.^[9]^" The former comprise molecular differences between patients and play a crucial role in both treatment and prognosis. The latter reflects the diversity within tumor cell subpopulations in primary tumors or metastatic diseases. Heterogeneity results from cancer cell-intrinsic traits (genome, epigenome, stemness, plasticity, migration, and proliferation capabilities) combined with microenvironmental factors (hypoxia, vascularization, stromal interactions, and the immune system).^[10]^

Tumor heterogeneity has been established as a fundamental factor in disease progression and metastatic spread. Gene profile comparisons between primary cancer cells and secondary lesions revealed significant heterogeneity, which was linked to organotropism and site-specific metastatic progression. In metastatic disease, differences were found between early and late metastatic cells.^[11]^ These differences are of utmost relevance when comparing untreated and treated patients. The metastatic profile of untreated patients revealed predominantly polyclonal seeding, which is a rare event in treated patients. Furthermore, specific mutations appear to be selected by therapy and contribute to drug resistance.

The discordance in estrogen receptor, progesterone receptor, and HER2 status between primary and metastatic lesions is well described. Although the conversion from positive to negative is more common, both conversions are relevant. In a systematic review performed by Schrijver et al, discordance percentages of 19.3% for estrogen receptor, 30.9% for progesterone receptor, and 10.3% for HER2 status, found.^[12]^ Modification of the therapeutic plan based on metastatic biopsy were more frequent when there was gain of receptor status, occurring in 14% to 62% of converted ER or PR receptors and 67% for HER2 status. However, the long-term effects of this modification have not yet been reported.^[12]^

This case report illustrates the relevance of the molecular characterization of metastatic progression to better tailor subsequent therapeutic lines. After 8 years of treatment, progression under first line metastatic endocrine therapy showed a relevant molecular feature – positivity for HER2. The gain in HER2 expression is in line with a recent retrospective study showing a higher percentage of patients with HER2 expression (10.36%) in metastatic lesions than in patients with loss of HER2 expression (5%).

According to the National Comprehensive Cancer Network Guidelines,^[13]^ HER2 positive metastatic breast cancer has a formal indication for palliative chemotherapy with taxanes plus double anti-HER2 blockage (trastuzumab plus pertuzumab) according to the PERUSE Trial. If progression was observed under this regimen, subsequent treatment with TDM-1 was administered according to the EMILIA trial. Regarding the gain of HER2 expression, the patient underwent treatment with docetaxel plus double anti-HER2 blockage and subsequent TDM-1 for 13 months with stable disease. However, rapid progression under HER2 blockage, together with previously documented tumoral heterogeneity, led to new tissue sampling for lung metastasis. Once more, a distinct disease was found to be triple-negative for metastatic progression. Facing triple-negative metastatic breast cancer in a woman with germline BRCA mutations, the National Comprehensive Cancer Network guidelines support the use of a platinum regimen as one of the preferred options. Therefore, systemic palliative therapy with carboplatin was initiated, allowing more than 3 months of stable disease until further progression. After progression under 5th metastatic line, targeted therapy with olaparib was initiated according to the results of the OlympiAD phase III clinical trial. The OlympiAD trial compared monotherapy with olaparib to standard therapy in germline BRCA mutated HER2 negative metastatic breast cancer with fewer than two previous chemotherapy regimens for metastatic disease.^[7]^

The poly (ADP-ribose) polymerase (PARP) family plays a crucial role in the regulation of transcription, apoptosis, and the DNA damage response. PARP 1 has poly (ADP-ribose) activity, which is responsible for the recruitment of other repair proteins to promote DNA single-strand break repair. PARPi competes for the NAD+ active site on PARP molecules, blocking its action and subsequent repair. In BRCA-mutated cancers, PARPi have been shown to be very effective in homologous recombination repair deficient tumors, such as BRCA mutations, due to a synthetically lethal interaction.^[14,15]^According to the National Authority of Medicines and Health Products, olaparib is approved for monotherapy in BRCA-mutated locally advanced or metastatic breast cancer HER2 negative previously treated with an anthracycline-or taxane-based regimen in the (neo)adjuvant or metastatic setting. Monotherapy with olaparib 300 mg twice daily demonstrated an increase in the median progression-free survival of 2.8 months with a 42% lower risk of disease progression or death. Based on these results, the patient was initiated on olaparib 300 mg twice daily for 28 days until the disease progressed. This tailored treatment approach allowed for an overall survival of 108 months with an excellent quality of life.

## 4 Conclusion

The clinical relevance of the discordant expression of estrogen, progesterone, and HER2 between primary and secondary lesions has been investigated in recent years. Some studies have suggested that concordant disease with maintenance of hormonal receptor expression has better outcomes than discordance with loss of receptors to a triple-negative disease.^[16]^ Regarding HER2 status, the results of the recent ChangeHER trial suggested that the gain of HER-2 expression in metastatic breast cancer was associated with better prognosis and more favorable overall survival.^[17]^ Despite, evidence on the impact of receptor conversion on clinical behavior and treatment outcomes is still scarce.

This case report illustrates the clinical relevance of re-assessing receptor status during each progression because it provides insight into the pattern of hormone receptor conversion and allows for better treatment tailoring. Prospective clinical trials are needed to study the clinical implications of hormone receptor conversion dynamics.

## Author contributions

Alexandra Mesquita was the medical oncologist responsible for clinical assessment and treatment, data assessment, and manuscript review.

Diogo J. Silva was responsible for literature review, data assessment, manuscript writing, and reviewing.

Gonçalo Miranda was responsible for anatomopathological analysis, imaging production and manuscript review.

**Conceptualization:** Diogo Silva.

**Data curation:** Diogo Silva, Gonçalo Miranda.

**Formal analysis:** Alexandra Mesquita.

**Supervision:** Alexandra Mesquita.

**Validation:** Alexandra Mesquita.

**Writing – original draft:** Diogo Silva.

**Writing – review & editing:** Alexandra Mesquita, Diogo Silva.
